# The rise and future of peptide-based antimicrobials

**DOI:** 10.71150/jm.2510002

**Published:** 2026-01-30

**Authors:** Hyo Jung Kim

**Affiliations:** College of Pharmacy, Woosuk University, Wanju 55338, Republic of Korea

**Keywords:** peptide antibiotics, antimicrobial resistance, synthetic biology, artificial intelligence, drug delivery systems

## Abstract

The escalating threat of antimicrobial resistance has renewed global interest in peptide-based antibiotics as adaptable and effective alternatives to conventional small molecules. Peptides possess diverse mechanisms of action, high target specificity, and structural flexibility, which collectively limit the emergence of resistance. This review outlines recent advances spanning the discovery, optimization, and application of peptide antibiotics, from their biological origins and structural classifications to emerging strategies involving artificial intelligence, synthetic biology, and modern delivery technologies. Peptide antibiotics can be categorized by origin as natural, semi-synthetic, or fully synthetic, and further organized by structural class such as α-helical, β-sheet, cyclic, and extended forms. They are also grouped by function into membrane-targeted and non-membrane-targeted types. These classification schemes are not only descriptive but also critical for understanding the therapeutic potential of peptides, as each category presents distinct advantages and engineering challenges that influence stability, specificity, and overall clinical performance. Advances in artificial intelligence, synthetic biology, and continuous manufacturing are reshaping how peptide drugs are designed and produced, while innovations in drug delivery systems are addressing critical issues of stability and bioavailability. Together, these developments are laying the foundation for a new generation of peptide-based therapeutics capable of meeting the evolving challenges of antimicrobial resistance.

## Introduction

The antibiotic era commenced in the early 1900s with the introduction of salvarsan by Paul Ehrlich, who conceptualized the “magic bullet.” This concept referred to a compound capable of selectively targeting pathogens while sparing the host (Ehrlich, 1913; Strebhardt and Ullrich, 2008). Since then, numerous classes of antibiotics have been developed, fueling optimism that infectious diseases could be completely defeated. However, this vision has proven overly optimistic. Over the past century, bacteria have continually evolved, leading to the emergence of antibiotic-resistant strains that compromise the efficacy of existing therapeutics. As antimicrobial resistance spreads globally, many small-molecule antibiotics once considered revolutionary have become increasingly ineffective (Giacomini et al., 2021; Uddin et al., 2021). One of the key reasons for this failure lies in the broad-spectrum nature of most traditional antibiotics. While broad-spectrum activity can be advantageous for treating a wide range of infections, it also exerts strong selective pressure on microbial populations. This accelerates bacterial evolution, driving the rapid emergence and dissemination of resistance mechanisms. As a result, the very strength of small-molecule antibiotics has become their greatest weakness.

Peptide antibiotics have emerged as a promising alternative to overcome these limitations (Vrbnjak and Sewduth, 2024; Zheng et al., 2025). Unlike small molecules, peptide-based therapeutics can target novel mechanisms of action. They also exhibit high target specificity, which significantly reduces the likelihood of resistance development. Their structural diversity enables them to interact with bacterial membranes, enzymes, and signaling pathways in ways that small molecules cannot easily achieve (Baral and Choi, 2025; Verma et al., 2021; Wu et al., 2022). Moreover, their specificity minimizes collateral damage to the host microbiota, an important consideration for preserving overall health.

Recent advances in artificial intelligence (AI) and in silico design have further accelerated peptide drug discovery. AI-driven models can rapidly explore vast sequence spaces and optimize peptides for potency, stability, and selectivity, streamlining the design process. In parallel, the development of solid-phase peptide synthesis and other manufacturing techniques has steadily reduced production costs, making peptide-based therapeutics increasingly feasible for clinical applications (Dong et al., 2025a; Lin et al., 2023b; Mwangi et al., 2023; Yan et al., 2022).

Despite these advantages, challenges remain. Peptides are inherently susceptible to proteolytic degradation within the body, leading to short half-lives and reduced efficacy. Effective delivery is another critical hurdle, as peptides often struggle to reach their targets at therapeutic concentrations. Furthermore, although costs are decreasing, peptide production remains more expensive than that of small-molecule drugs, posing barriers to widespread commercialization. To fully realize the potential of peptide antibiotics, advances in peptide design and modification, drug delivery systems (DDS), and formulation strategies must converge. By integrating these disciplines, it may be possible to create a new generation of peptide therapeutics capable of addressing the global crisis of antibiotic resistance.

## Origin-based Classification of Peptide Antibiotics

Peptide antibiotics comprise a diverse class of molecules with different origins, structures, and mechanisms of action. They are broadly divided into three groups: naturally occurring peptides, semi-synthetic derivatives, and fully synthetic or engineered peptides, reflecting a progression from natural evolutionary products to rationally designed therapeutics.

Naturally occurring peptides form the foundation of this class and are produced by microorganisms, plants, or animals for ecological competition. They fall into two main biosynthetic groups: ribosomally synthesized and post-translationally modified peptides (RiPPs) and non-ribosomal peptides (NRPs). RiPPs are made by ribosomes as precursor peptides and then enzymatically modified to become active. Class I bacteriocins (lantibiotics)-such as nisin and lacticin 3147-target lipid II and are predominantly active against Gram-positive bacteria (Morgan et al., 2005). Class II bacteriocins, exemplified by pediocin PA-1, display receptor-dependent, highly species-specific killing (Goldbeck et al., 2021). Other RiPPs, such as sactipeptides and thiopeptides (subtilosin A, thiostrepton), contain distinctive sulfur-carbon bonds and thiazole rings. These structural motifs enhance molecular stability and diversify their mechanisms (Cao et al., 2021; Wenski et al., 2022; Zhong et al., 2023). NRPs are assembled by non-ribosomal peptide synthetase (NRPS) enzyme complexes, which incorporate unusual amino acids and modifications (Strieker et al., 2010). Examples include several distinct subclasses. Lipopeptides such as daptomycin and polymyxin B/E disrupt bacterial membranes. Glycopeptides including vancomycin, teicoplanin, and dalbavancin inhibit cell wall synthesis. In contrast, sideromycins such as albomycin exploit bacterial iron uptake pathways (Cafiso et al., 2012; Dassonville-Klimpt and Sonnet, 2020; Taylor and Moreira, 2025).

The second major category consists of semi-synthetic derivatives, in which naturally derived scaffolds are chemically modified to enhance pharmacological properties. These modifications can improve pharmacokinetics, increase membrane-binding affinity, expand antimicrobial spectrum, or reduce toxicity. Examples include optimized lipoglycopeptides and next-generation polymyxin analogs that build upon natural backbones while overcoming some of their clinical limitations (Jiang et al., 2021; Slingerland et al., 2022).

**Fig. 1.**
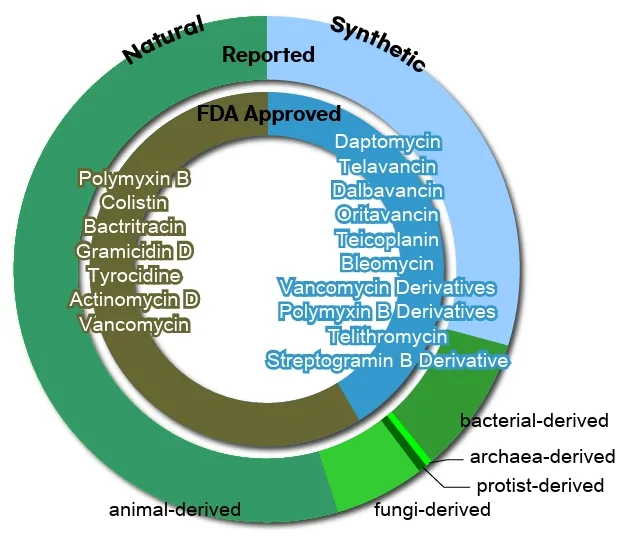
Landscape of approved and reported peptide antibiotics. The inner diagram represents FDA-approved peptide antibiotics (seven natural and ten synthetic agents) and the outer diagram shows reported peptides with documented antimicrobial activity, most of which are animal- or bacterial-derived. Blue shades indicate synthetic or semi-synthetic peptides, whereas green shades correspond to naturally derived peptides of bacterial or animal origin. This classification highlights the dominance of naturally derived scaffolds among approved drugs and the growing diversity of synthetic analogs under investigation. The data are from the Antimicrobial Peptide Database (APD6) (Wang et al., 2025b).

Finally, fully synthetic and engineered peptides represent the frontier of modern drug design. These compounds may be entirely designed de novo, using rational design principles to tune charge distribution, amphipathicity, and secondary structure for optimal activity and selectivity (Fjell et al., 2011). In other cases, naturally occurring peptide scaffolds are deliberately engineered to improve their performance through techniques such as stapling, lipidation, or incorporation of non-natural amino acids (Dean et al., 2024; Walensky and Bird, 2014). Emerging strategies further broaden this landscape. They include hybrid conjugates that combine multiple functional domains, peptide-PNA conjugates for targeting genetic material, and targeted delivery peptides fused to receptor-binding ligands for enhanced specificity (Fig. 1).

## Structure-based Classification of Peptide Antibiotics

Antibacterial peptides can be usefully organized into four structural classes-α-helical, β-sheet, cyclic, and extended-each with characteristic structure-function relationships, strengths, and liabilities relevant to drug design.

α-Helical peptides are typically linear, cationic sequences that become amphipathic helices upon encountering membranes. This induced folding exposes a contiguous hydrophobic face opposite clusters of Lys, Arg, and His. The resulting amphipathic structure promotes adsorption to anionic bacterial surfaces and subsequent insertion. Representative examples include LL-37 and gramicidin A, both of which exemplify membrane-active α-helical peptide antibiotics (Gesell et al., 1997; Wang, 2008). Their dominant mechanisms involve membrane disruption-via carpet, toroidal pore, or barrel-stave-like models-though intracellular effects are reported for some members. Advantages include straightforward synthesis, clear sequence-activity rules, and broad activity against both Gram-positive and Gram-negative bacteria. However, the main liabilities include hemolysis and serum binding, which can erode selectivity and potency. Rational design focuses on helix-wheel balance (hydrophobic moment), net charge (often +3 to +6), end-capping, and stability tweaks such as D-amino acids or acylation (Dathe et al., 2001; Lu et al., 2020).

β-Sheet peptides are compact scaffolds stabilized by one or more disulfide bonds that lock β-hairpins or short sheets. In short peptides, β-hairpin conformations are generally unstable due to conformational strain and entropic flexibility. To stabilize such structures, physical constraints are often introduced. These include β-turn motifs that promote compact folding, disulfide bridges, aromatic stacking, or ionic interactions that strengthen the β-sheet framework (Fahrner et al., 1996; Laederach et al., 2002). This constrained topology yields high protease resistance and preserves activity under physiological salt conditions. Many exhibit potent membrane permeabilization; others bind defined cell-envelope ligands, including LPS or Lipid II. Typical challenges are redox sensitivity (disulfide reduction in intracellular milieus) and aggregation. Engineering strategies tune the number and placement of disulfides, modulate surface charge patches, or replace cystines with non-reducible linkages to maintain the β-hairpin geometry (Li et al., 2021; Shi et al., 2018; Sivanesam et al., 2016).

Cyclic antimicrobial peptides are macrocyclic scaffolds in which backbone or side-chain linkages impose a closed topology. Macrocyclization lowers conformational entropy and typically enhances target affinity, selectivity, and metabolic stability. These agents often act through specific target engagement, such as binding the pyrophosphate of Lipid II, hijacking outer-membrane receptors, or calcium-dependent membrane depolarization (Ball et al., 2004; Economou et al., 2013). Cyclic scaffolds excel in potency and half-life but can pose synthetic and formulation challenges due to noncanonical linkages or hydrophobic tails. Medicinal chemistry strategies include tuning ring size and flexibility, positioning crosslinks, and modifying lipid appendages to balance potency and solubility (Guo et al., 2023; Koehbach et al., 2024; Tan et al., 2024).

Extended peptides lack persistent secondary structure and are frequently enriched in Trp, Pro, or Arg (Panteleev et al., 2024; Rozek et al., 2000). Their flexibility enables multimodal action: initial membrane association followed by entry and interference with intracellular processes (e.g., nucleic acid binding, ribosome or chaperone disruption). This plasticity can promote broad-spectrum activity and resistance avoidance but raises risks of nonspecific cytotoxicity and rapid clearance. Design focuses on optimal length and Trp-Arg compositions, terminal capping, partial D-substitution, or backbone modifications to enhance stability while steering interactions toward desired intracellular targets (Han et al., 2021).

**Fig. 2.**
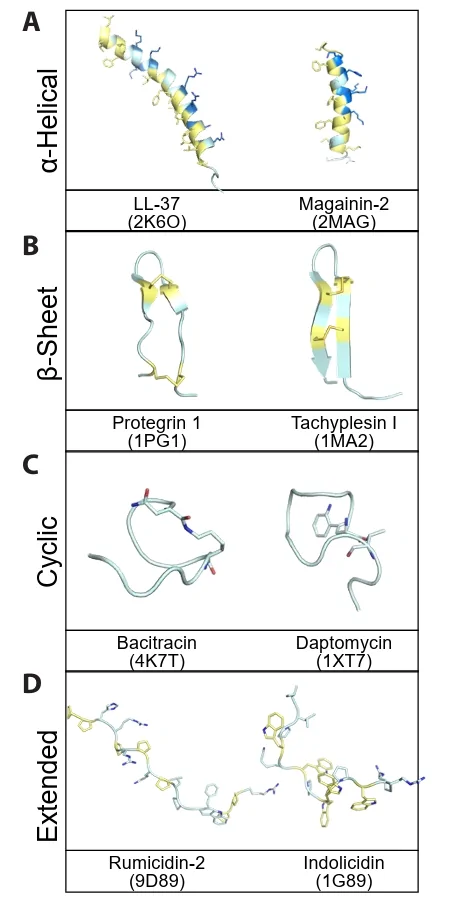
Representative peptide antibiotics with experimentally resolved structures. (A) α-Helical peptides typically exhibit amphipathic character, with one face composed of hydrophobic residues shown in yellow and the opposite enriched in positively charged residues shown in blue. (B) β-Sheet peptides often contain disulfide (colored in yellow) or covalent linkages that stabilize the overall structure. (C) Cyclic peptides are cross-linked through various intramolecular bonds; the examples shown include Lys-Asn and kynurenine-Thr linkers. (D) Extended peptides frequently feature repetitive amino acid motifs, exemplified here by sequences rich in Pro and Trp residues. All structures were obtained from the Protein Data Bank (PDB; IDs in parentheses) and visualized using PyMOL. The color scheme highlights hydrophobic (yellow) and cationic (blue) residues to emphasize amphipathic architecture.

Viewed together, these classes provide complementary routes to antibacterial efficacy: membrane-centric disruption (α-helical, many β-sheet, and lipidated cyclic) and target-centric recognition (cyclic, some β-sheet, and extended). Effective development hinges on matching scaffold topology to mechanism, then mitigating class-specific liabilities through sequence and topology engineering (Fig. 2).

## Mechanism-based Classification of Peptide Antibiotics

Peptide antibiotics can be broadly classified into membrane-targeting and non-membrane-targeting types, reflecting two fundamental modes of antimicrobial action (Mahlapuu et al., 2016). Membrane-targeting peptides act by perturbing or disrupting bacterial membranes, which are rich in negatively charged phospholipids such as phosphatidylglycerol and cardiolipin (Chen et al., 2018). Their cationic and amphipathic nature enables electrostatic attraction to bacterial surfaces followed by hydrophobic insertion into lipid bilayers. This rapid, largely nonspecific mechanism makes resistance uncommon. Within this group, membrane-disruptive peptides physically damage the bilayer through mechanisms described by the carpet, barrel-stave, and toroidal-pore models (Melo et al., 2009). Although these models differ conceptually, most peptides display mixed behavior depending on concentration and membrane composition (Mangoni and Shai, 2009). Other peptides only permeabilize membranes transiently, as seen with temporins or cecropins, which create short-lived defects that alter membrane potential and facilitate uptake of other antibiotics. A third subgroup, membrane-binding modulators, mainly associates with the surface to alter properties such as curvature or fluidity. Defensins and hybrid peptides often act this way, sensitizing microbes to external stress while minimizing host toxicity (Schmidts et al., 2019).

Non-membrane-targeting peptides operate beyond the membrane, affecting intracellular or host-mediated pathways. Intracellular-targeting peptides translocate into the cytoplasm and inhibit vital processes like nucleic acid or protein synthesis. For example, proline-rich peptides such as PR-39 or Bac7 enter via transporter proteins and bind ribosomes or chaperones to block translation, whereas indolicidin interacts directly with DNA. These compounds are generally less cytotoxic to host cells (Krizsan et al., 2014; Seefeldt et al., 2016).

Immune-modulatory peptides, or host defense peptides, act indirectly by modulating the immune system. LL-37 exemplifies this dual activity, displaying antimicrobial effects while regulating cytokine release and wound healing. β-Defensins also participate in immune signaling by recruiting immune cells to infection sites. Another functional group, the quorum-sensing-interfering peptides, disrupts bacterial communication systems controlling biofilm formation and virulence. The RNAIII-inhibiting peptide (RIP) in *Staphylococcus aureus* is a well-known example that suppresses pathogenicity without promoting resistance (Hancock et al., 2012; Rios et al., 2016).

**Fig. 3.**
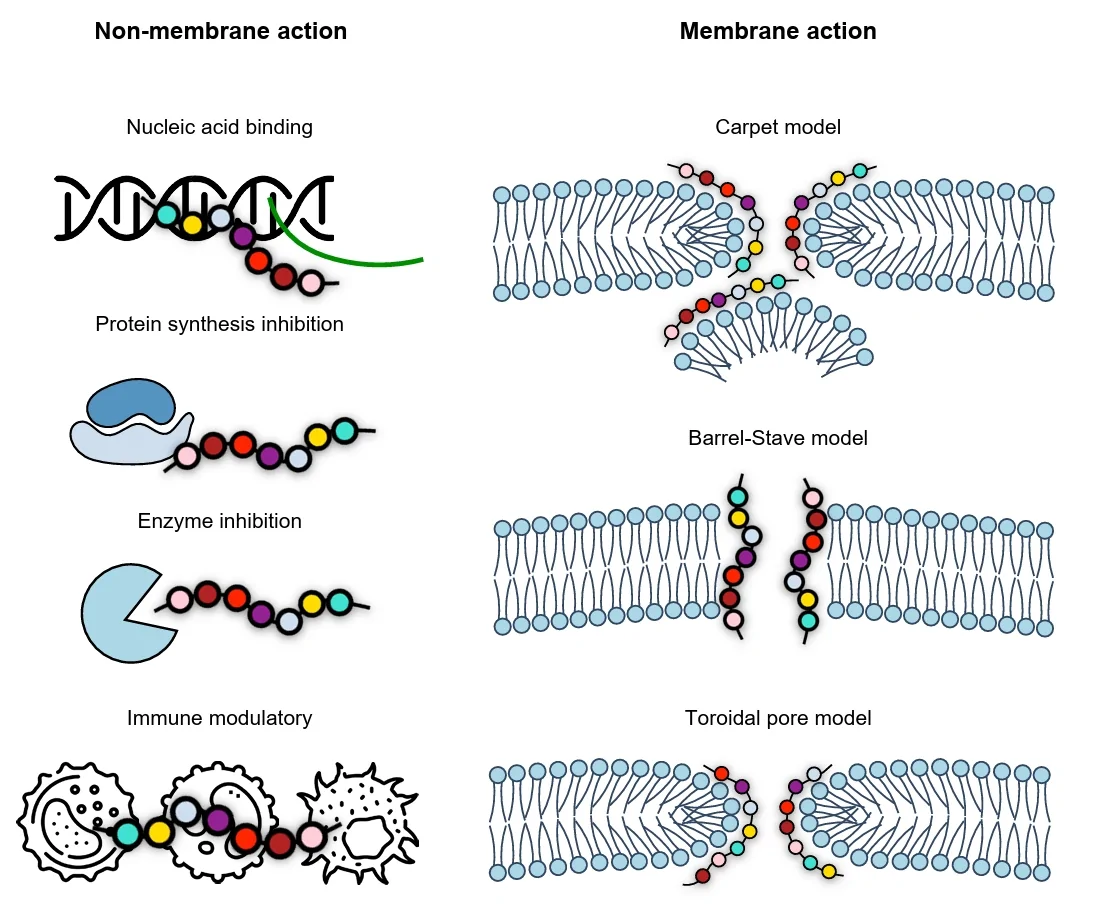
Mechanistic modes of action of peptide antibiotics. This schematic illustrates representative mechanisms by which peptide antibiotics exert antibacterial activity. (Left) Non-membrane-targeting mechanisms, including (i) inhibition of nucleic acid synthesis by direct binding to DNA or RNA, (ii) inhibition of protein synthesis through interaction with ribosomes or chaperones, (iii) interference with enzymatic activity, and (iv) modulation of immune or signaling pathways. (Right) Membrane-targeting mechanisms, comprising (i) the carpet model, in which peptides cover and destabilize the membrane surface, (ii) the barrel-stave model, where peptides insert to form transmembrane pores, and (iii) the toroidal-pore model, in which both peptides and lipid head groups participate in pore formation. Together, these mechanisms highlight the structural and functional diversity of peptide antibiotics and their ability to disrupt bacterial viability through both membrane and intracellular pathways.

Overall, membrane-targeting peptides provide fast, broad-spectrum activity, whereas non-membrane-targeting peptides offer more selective and often synergistic modes of action. Recognizing these distinctions clarifies the mechanistic landscape of antimicrobial peptides and guides rational design strategies to improve selectivity, stability, and therapeutic outcomes (Fig. 3).

## AI-Driven Design and Molecular Optimization Strategies for Peptide Antibiotics

AI offers a transformative solution by enabling rational, rapid exploration of peptide sequences within an astronomical search space, which for a peptide of length n can encompass up to 20n possible combinations. Instead of relying solely on trial-and-error, AI models can design novel peptides, predict their properties, and refine them through experimental feedback. This approach enables early identification and prioritization of promising candidates. As a result, both time and cost are reduced, while success rates increase. Deep generative models such as GANs, VAEs, and transformer-based, large language models (LLMs) have been used to create entirely new peptide sequences by learning patterns from known antimicrobial peptides. These models generate diverse and functionally relevant candidates far beyond what manual design can achieve.

In addition to generation, AI excels at predicting complex features such as antimicrobial potency, toxicity, and stability. By integrating sequence, structural, and experimental data, deep learning models can assess multiple traits simultaneously. This allows researchers to focus on the most viable molecules. Optimization is further advanced through reinforcement learning and Bayesian approaches, which iteratively refine sequences to enhance activity and reduce side effects. Importantly, laboratory results such as activity or toxicity data are fed back into AI systems to improve predictions, creating a continuous design-build-test-learn cycle.

Real-world successes illustrate this potential: AI platforms have narrowed libraries of hundreds of thousands of theoretical peptides down to a few dozen validated molecules. The LLAMP framework successfully screened millions of potential sequences, leading to the discovery of peptides with activities comparable to clinically approved antibiotics such as pexiganan. Similarly, the AMP-Diffusion generative model produced candidates validated in vitro and in vivo, including murine infection models. These results provide compelling evidence of translational potential (Jin et al., 2025; Torres et al., 2025; Wang et al., 2024, 2025c). Moreover, integration of computational design with cell-free biosynthesis has enabled rapid experimental screening, as demonstrated by the identification of broad-spectrum peptides from hundreds of AI-generated sequences (Pandi et al., 2023). Finally, the AMP-Designer platform exemplifies how large language models can drastically reduce development timelines, generating active, low-toxicity peptides within less than two weeks (Wang et al., 2025a). Several challenges remain, including limited datasets, experimental bias, and the difficulty of interpreting deep learning models. Nevertheless, the integration of AI with wet-lab research marks a paradigm shift. By combining generative design, predictive modeling, and iterative optimization, AI accelerates the development of next-generation peptide antibiotics to address the urgent threat of antimicrobial resistance.

## Synthetic Biology and Manufacturing Strategies

A major barrier to the clinical translation of peptide antibiotics is their high production cost and complexity. Unlike small-molecule drugs, peptides are larger and structurally complex, making them difficult and expensive to produce. Traditional solid-phase peptide synthesis (SPPS) is highly effective for research-scale synthesis but becomes prohibitively costly and labor-intensive for large-scale manufacturing, especially for longer sequences.

Synthetic biology offers a solution by enabling microbial production of peptide antibiotics using engineered hosts such as *Escherichia coli*, *Bacillus subtilis*, and *Streptomyces*. Through CRISPR-based genome editing and metabolic engineering, biosynthetic pathways can be optimized to channel cellular resources toward peptide production. This approach has been applied to both ribosomally synthesized peptides like lantibiotics and non-ribosomal peptides such as polymyxins and daptomycin. It has achieved significantly higher yields while reducing reliance on chemical synthesis.

**Fig. 4.**
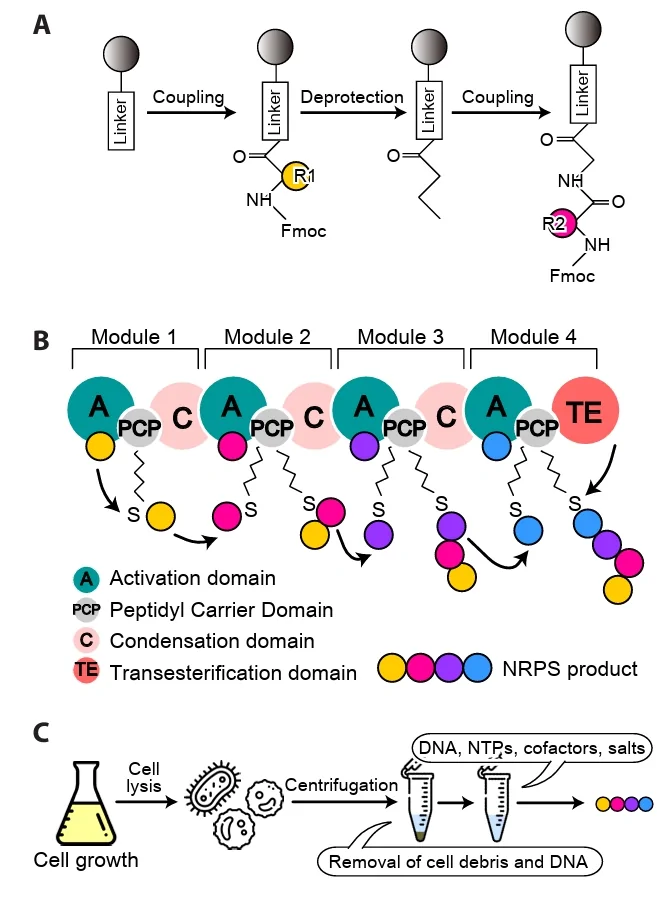
Representative approaches for peptide synthesis. This figure provides a simplified schematic of peptide synthesis methods. Together, these panels highlight the conceptual differences between chemical, enzymatic, and cell-free peptide synthesis strategies. (A) Classical solid-phase peptide synthesis (SPPS) illustrates the stepwise chemical coupling of amino acids, exemplified by the first two residues. (B) Non-ribosomal peptide synthetase (NRPS) pathway depicts the modular enzymatic assembly of peptide chains, where A, C, PCP, and TE denote adenylation, condensation, peptidyl carrier protein, and thioesterase domains, respectively. (C) Cell-free protein synthesis (CFPS) represents the in vitro translation-based production of peptides without living cells.

A major advantage of synthetic biology is its modular design capability. Non-ribosomal peptide synthetases (NRPS) can be reconfigured to produce novel analogs not found in nature, while synthetic gene circuits provide precise control over expression and post-translational modifications. For highly toxic peptides, cell-free protein synthesis (CFPS) provides an alternative platform. It enables rapid in vitro production and high-throughput screening of AI-designed peptide libraries, though cost optimization remains a challenge (Fig. 4).

Beyond synthesis, downstream processing such as purification and quality control is essential for cost reduction. Recent industrial advances, such as multicolumn countercurrent solvent gradient purification (MCSGP), have greatly improved efficiency. Continuous MCSGP improves process efficiency, with reported yield gains of ~21% and solvent reductions of up to 75% (Luca et al., 2020). Advances in continuous processing and automation are streamlining these steps, while AI-driven process optimization allows real-time adjustments of fermentation parameters to maximize yields. By integrating synthetic biology with smart automation, scalable and economically viable peptide manufacturing is becoming increasingly feasible.

In summary, combining engineered microbes, cell-free systems, and computational optimization offers a path toward cost-effective, large-scale production of peptide antibiotics. Synthetic biology has been applied to create novel peptide antibiotics by reconfiguring NRPS assembly lines, such as those producing daptomycin, to generate derivatives with improved stability and potency (Alanjary et al., 2019). Cell-free protein synthesis (CFPS) has also been combined with AI-driven design to rapidly synthesize and screen hundreds of candidate peptides, yielding dozens with strong antimicrobial activity (Pandi et al., 2023; Ratnayake et al., 2024). In addition, engineering NRPS modules to incorporate non-natural substrates has further expanded the diversity of bioengineered lipopeptides, providing new avenues for antibiotic development (Camus et al., 2023; Chen et al., 2023; Zhang et al., 2023).

## Delivery Systems and Strategies to Enhance In Vivo Stability

Peptide antibiotics show great promise but face significant barriers to clinical use due to rapid proteolytic degradation, short half-lives, and poor membrane permeability. Most peptides cannot be taken orally because they are destroyed in the gastrointestinal tract, requiring injection and frequent dosing. These issues increase treatment costs and raise the risk of systemic toxicity, highlighting the need for DDS that improve stability and ensure targeted delivery.

One strategy is to chemically modify peptides to resist degradation and extend circulation time. Methods such as D-amino acid substitution, cyclization, PEGylation, lipidation, and stapled peptide design enhance stability, solubility, and cell penetration while maintaining antimicrobial activity. Recently, AI-based prediction tools such as DeepCleave and PROSPER have been used to identify protease cleavage sites. In parallel, structure prediction models like AlphaFold2 and ESMFold predict 3D conformations to guide modifications such as cyclization and stapling (Fang et al., 2023; Li et al., 2020a, 2020b; McDonald et al., 2023; Song et al., 2012). These models allow rational design of more stable peptides before costly experimental testing.

Another approach involves nanocarrier-based delivery systems, which protect peptides from enzymatic breakdown and enable controlled release. Liposomes, polymeric nanoparticles, and solid lipid nanoparticles have been used to encapsulate peptides and improve their therapeutic concentrations at infection sites. To streamline this process, machine learning frameworks such as DeepChem and Gaussian Process Optimization (GPO) are being applied to predict and optimize nanocarrier parameters, including particle size, surface charge, and release profiles (Dong et al., 2025b; Esmaeilpour et al., 2025; Gao et al., 2024). In parallel, TorchMD and OpenMM molecular dynamics simulations model interactions between peptides and carrier materials, helping researchers refine designs virtually before production (Doerr et al., 2021; Eastman et al., 2024). These carriers can also be engineered for targeted delivery. Tools such as AlphaFold2-Multimer and AutoDock Vina assist in ligand-receptor mapping to select effective targeting molecules, including bacterial receptor ligands or cell-penetrating peptides (CPPs). This strategy enhances selectivity and minimizes off-target effects (Banhos Danneskiold-Samsøe et al., 2024; Rentzsch and Renard, 2015).

Stimuli-responsive systems represent the next generation of DDS, releasing peptides only under specific conditions like acidic pH or bacterial enzyme activity, improving safety and reducing dosing frequency. Preclinical studies, including liposomal formulations of polymyxins and vancomycin, have already demonstrated enhanced efficacy and reduced toxicity compared to free drugs (Antoniou et al., 2021; Zhang et al., 2021; Zhou et al., 2024).

Integrating DeepCleave- and AlphaFold2-guided peptide design with DeepChem-optimized nanocarriers enables a closed-loop development pipeline. This combined computational and experimental strategy offers a path to overcoming stability and bioavailability barriers, accelerating the translation of peptide antibiotics into clinically viable therapies for multidrug-resistant infections.

## Clinical Trials and Commercialization Prospects

The clinical translation of peptide antibiotics has gained increasing attention as antimicrobial resistance continues to rise. Several peptide-based therapeutics have already reached the market, while many others are advancing through the clinical pipeline, demonstrating both the promise and the challenges of bringing these drugs from bench to bedside.

Among the approved agents, daptomycin, a lipopeptide antibiotic, is widely used for the treatment of complicated Gram-positive infections, including those caused by methicillin-resistant *S. aureus* (MRSA) (Fiore et al., 2025). Similarly, polymyxin B and colistin, both naturally derived polypeptides, remain essential last-line therapies for multidrug-resistant Gram-negative bacteria (Nang et al., 2021). In addition, telavancin, a semi-synthetic lipoglycopeptide, has been approved for indications such as complicated skin and soft tissue infections and MRSA-associated pneumonia (Wenzler and Rodvold, 2015). These examples highlight that peptide-based antibiotics are already part of the current therapeutic landscape.

In the development pipeline, several promising antimicrobial peptide candidates are advancing through clinical stages. PLG0206 (Peptilogics) is a synthetic peptide designed to treat periprosthetic joint infections (Huang et al., 2022). It has successfully completed Phase I trials and has received both Orphan Drug and Qualified Infectious Disease Product (QIDP) designations from the U.S. FDA, highlighting its potential clinical impact. Another notable candidate is murepavadin, a first-in-class peptide that targets outer-membrane proteins of *Pseudomonas aeruginosa*. It is currently under Phase III clinical trials (Martin-Loeches et al., 2018; Srinivas et al., 2010). Beyond these examples, multiple AMP candidates are in earlier stages of clinical testing, targeting diverse pathogens and indications ranging from wound infections to respiratory diseases.

Despite these advances, the commercialization of peptide antibiotics faces several hurdles. Production costs remain high compared to small-molecule antibiotics, and ensuring adequate stability and targeted delivery in vivo is still a challenge (Bucataru and Ciobanasu, 2024; Szymczak and Szczurek, 2023). Large-scale clinical trials demand substantial investment and careful design. They must demonstrate not only efficacy but also safety and cost-effectiveness. However, innovations in synthetic biology, AI-driven peptide design, and advanced DDS are expected to reduce these barriers by streamlining development and lowering manufacturing costs.

In summary, the current landscape shows a growing number of peptide antibiotics transitioning from discovery to clinical reality. With successful examples such as daptomycin and colistin already in clinical use and next-generation candidates like PLG0206 and murepavadin progressing through trials, the field is moving steadily toward broader commercialization. These developments highlight the critical role of peptide therapeutics as part of the global strategy to combat multidrug-resistant infections.

## Concluding Remarks and Future Perspectives

AI-driven technologies are poised to accelerate peptide antibiotic discovery and optimization. Deep learning models such as AlphaFold2 and ESMFold enable high-resolution structure prediction, guiding the rational modification of peptides for enhanced stability and potency (Jumper et al., 2021; Lin et al., 2023a). Generative models like ProtGPT2 and AMP-GAN can rapidly explore vast sequence spaces to identify novel candidates. In parallel, predictive tools like DeepCleave and PROSPER identify protease cleavage sites to improve in vivo durability. Combined with computational frameworks like DeepChem and TorchMD, AI can also optimize nanocarrier formulations and simulate drug-carrier interactions. These capabilities reduce reliance on costly trial-and-error experiments (Doerr et al., 2021; Song et al., 2012; Yi et al., 2018). This closed-loop, in silico-to-in vitro workflow has the potential to drastically shorten development timelines while lowering overall costs.

Beyond individual technologies, global collaboration will play an increasingly important role in realizing the full potential of peptide antibiotics. International organizations such as the World Health Organization (WHO), the Centers for Disease Control and Prevention (CDC), and the Global Antibiotic Research & Development Partnership (GARDP) are well positioned to lead global coordination. They can facilitate data-sharing frameworks and standardized evaluation protocols. Furthermore, open-access AI platforms for peptide design and resistance monitoring could accelerate innovation and improve coordination among research communities. In parallel, adaptive regulatory pathways that integrate real-world evidence and flexible clinical trial designs will be critical to support the efficient and safe translation of novel peptides into clinical use.

Despite these opportunities, challenges remain. AI algorithms still face issues of transparency and data bias, which can limit their reliability. High manufacturing costs and the need for large-scale clinical validation also remain significant obstacles. Furthermore, the rapid evolution of bacterial resistance demands equally rapid innovation to stay ahead. Addressing these challenges will require coordinated efforts across disciplines, industries, and governments.

Looking to the future, the convergence of AI, synthetic biology, and advanced DDS holds the potential to revolutionize peptide antibiotic development. In the next decade, these integrated technologies could enable personalized peptide therapeutics, real-time global resistance monitoring, and scalable manufacturing pipelines. With sustained investment and international cooperation, peptide antibiotics could evolve from niche therapies into a cornerstone of the global strategy against multidrug-resistant infections. This shift may ultimately shape a new era in antimicrobial drug development.
